# Cortisol Metabolism in Carp Macrophages: A Role for Macrophage-Derived Cortisol in M1/M2 Polarization

**DOI:** 10.3390/ijms21238954

**Published:** 2020-11-25

**Authors:** Magdalena Maciuszek, Katarzyna Klak, Leszek Rydz, B. M. Lidy Verburg-van Kemenade, Magdalena Chadzinska

**Affiliations:** 1 Department of Evolutionary Immunology, Institute of Zoology and Biomedical Research, Faculty of Biology, Jagiellonian University, Gronostajowa 9, 30-387 Krakow, Poland; magdalena.maciuszek@doctoral.uj.edu.pl (M.M.); katarzyna.klak@doctoral.uj.edu.pl (K.K.); 2 Chair of Medical Biochemistry, Faculty of Medicine, Jagiellonian University Medical College, Kopernika 7, 31-034 Krakow, Poland; leszek.rydz@uj.edu.pl; 3 Cell Biology and Immunology, Wageningen University, P.O. Box 338, 6700AH Wageningen, The Netherlands; lidy.vankemenade@outlook.com

**Keywords:** macrophage polarization, IFN-γ + LPS, IL-4/13B, cortisol, 11β-HSD, GR, carp, teleost fish

## Abstract

Macrophages are crucial not only for initiation of inflammation and pathogen eradication (classically polarized M1 macrophages), but also for inflammation inhibition and tissue regeneration (alternatively polarized M2 macrophages). Their polarization toward the M1 population occurs under the influence of interferon-γ + lipopolysaccharide (IFN-γ + LPS), while alternatively polarized M2 macrophages evolve upon, e.g., interlukin 4 (IL-4) or cortisol stimulation. This in vitro study focused on a possible role for macrophage-derived cortisol in M1/M2 polarization in common carp. We studied the expression of molecules involved in cortisol synthesis/conversion from and to cortisone like 11β-hydroxysteroid dehydrogenase type 2 and 3. (11β-HSD2 and 3) and 11β-hydroxylase (CYP11b), as well as the expression of glucocorticoid receptors (GRs) and proliferator-activated receptor gamma (PPARγ) in M1 and M2 macrophages. Lastly, we analyzed how inhibition of these molecules affect macrophage polarization. In M1 cells, upregulation of gene expression of GRs and 11β-HSD3 was found, while, in M2 macrophages, expression of *11β-hsd2* was upregulated. Moreover, blocking of cortisol synthesis/conversion and GRs or PPARγ induced changes in expression of anti-inflammatory interleukin 10 (IL-10). Consequently, our data show that carp monocytes/macrophages can convert cortisol. The results strongly suggest that cortisol, via intracrine interaction with GRs, is important for IL-10-dependent control of the activity of macrophages and for the regulation of M1/M2 polarization to finally determine the outcome of an infection.

## 1. Introduction

Macrophages are crucial leukocytes for both innate and adaptive immune responses [1]. They form a heterogeneous population of cells, among which resident and inflammatory/migratory cells are distinguished. Moreover, during inflammation, macrophages play different roles. Classically, polarized M1 macrophages are involved in the initiation and development of inflammation, while M2 macrophages are important during eradication of inflammation and tissue regeneration/wound healing [2]. Therefore, the macrophage polarization process is crucial for a proper course of inflammation to allow elimination of pathogens and/or damaged cells without excessive damage to healthy tissues of the host.

In mammals, macrophage polarization toward the M1 population occurs under the influence of interferon-γ (IFN-γ), lipopolysaccharide (LPS), and tumor necrosis factor α (TNF-α) [2]. M1 macrophages produce pro-inflammatory cytokines (including interleukin 1β (IL-1β), interleukin 12 (IL-12), TNF-α), chemokines (CXCL9–11, CCL2–5), reactive oxygen species (ROS) and nitric oxide (NO) [3]. In this way, a proinflammatory cascade is initiated leading to the elimination of the pathogen [4]. Crucial transcription factors involved in classical macrophage polarization are signal transducer and activator of transcription 1 (STAT1) and IFN regulatory factor 5 (IFR5), which initiate gene expression of proinflammatory mediators such as *inos*, *il-12*, *il-23*, and *tnf-α*. These mediators enhance the biocidal function of macrophages [5].

In turn, mammalian M2 macrophages are more diverse and, depending on the stimulant, they can polarize toward several subpopulations (M2a, b, and c). Alternatively, polarized M2a macrophages evolve upon interleukin 4 (IL-4) or interleukin 13 (IL-13) stimulation, show high activity of arginase, and produce polyamine, interleukin 10 (IL-10), and antagonists of the interleukin 1 receptor (IL-1Ra). They are involved in Th2 responses, active in allergic reactions and in parasite killing. M2b macrophages are induced by a combined exposure to not only immune complexes and Toll-like receptors (TLRs) or IL-1Ra. They release IL-10, but also proinflammatory cytokines such as TNF-α, IL-1, and IL-6; therefore, they are involved in activation of Th2 lymphocytes and in immunoregulation. In turn, M2c macrophages polarize upon treatment with IL-10 or with stress-induced glucocorticoid hormones, e.g., cortisol. M2c macrophages produce IL-10 and transforming growth factor-β (TGF-β) and are associated with immune suppression and tissue remodeling [3]. Pivotal transcription factors involved in alternative polarization are STAT6, IRF4, and peroxisome proliferator-activated receptor gamma (PPARγ), and their activation leads to increased expression of M2 markers (arginase 1, Fizz1, Ym1, and CD206) [5].

Over the last decennia, it has become clear that the neuroendocrine and immune systems coordinate their actions in a bidirectional fashion, involving neuroendocrine receptors on immune cells and cytokine receptors on vital cells of the neuroendocrine system. This interaction is evolutionarily conserved and involves messaging by cytokines to the brain, and hormones, neuropeptides, and neurotransmitters to the endocrine system. These molecules can furthermore be produced in both systems [6].

Interestingly, Thieringer and colleagues [7] suggested that, in addition to the cortisol produced upon stress in the adrenal glands, macrophage-derived cortisol may also be important for the regulation of macrophage polarization. They demonstrated that human monocyte incubation with IL-4 or IL-13 induced activity of the 11β-hydroxysteroid dehydrogenase type 1 (11β-HSD1). 11β-HSD1 is a nicotinamide adenine dinucleotide (NADPH)-dependent enzyme, converting the intrinsically inert cortisone and 11-dehydrocorticosterone into the active glucocorticoids, cortisol, and corticosterone, respectively [8]. It must be mentioned that, both in mammals and fish, the intracellular level of cortisol is regulated by multiple enzymes [9,10]. In addition to 11β-HSD1, NAD^+^-dependent 11β-HSD2 can convert cortisol to cortisone [8], while 11β-hydroxylase (CYP11b) can catalyze the conversion of 11-deoxycortisol to cortisol [9]. The latter reaction is limited by the expression of steroidogenic acute regulatory protein (StAR), which regulates cholesterol transfer [11]. In humans, loss of the activity of 11β-HSD1 results in a disorder termed cortisone reductase deficiency (CRD), which results in failure to regenerate the active cortisol from cortisone. CRD is manifested by adrenocorticotropic hormone (ACTH)-mediated adrenal hyperandrogenism, which in males is associated with precocious pseudopuberty. Females with CRD, in adolescence and early adulthood, suffer from hirsutism, oligomenorrhea, and infertility [12]. In turn, deficiency of 11β-HSD2 causes the syndrome of apparent mineralocorticoid excess (AME), which is characterized by hypertension, hypokalemia, and suppressed plasma renin and aldosterone levels (for a review, see White [13]). Although glucocorticoids are commonly prescribed for their anti-inflammatory actions, to date, relatively few studies addressed the involvement of 11β-HSDs in glucocorticoid-mediated immune functions, while, to our best knowledge, immune-related changes in CRD or AME disorders have not been systematically studied.

Interestingly, the presence of StAR was found in murine macrophages [14], human monocytes, and the THP-1 macrophage cell line [15,16,17], and its expression was regulated by cytokines, PPARγ, or by the retinoid X receptor (RXR) agonist [14,15,18]. For example, Ma and colleagues [14] showed that TNF-α and IFN-γ reduced StAR expression, while TGF-β increased its expression in RAW264.7 macrophages. In fish, gene expression of StAR, CYP11b, and 11β-HSD2 was confirmed in endocrine tissues/cells [10], while 11β-HSD1 was not found in teleosts. Instead, they possess 11β-HSD3, which most probably is the ancestor and paralog of 11β-HSD1 [19,20]. However, little is known about the functionality of this enzyme.

Until now, several papers showed classical polarization of fish macrophages upon stimulation with LPS, IFN-γ + LPS, zymosan or *Trypanosoma borreli* parasites, while alternative polarization was observed upon treatment with IL-4/13, prostaglandin E2, and cAMP [21,22,23,24,25,26,27,28,29,30,31]. Moreover, recently, we revealed that exogenous cortisol affects the polarization status of monocytes/macrophages in carp [32].

In this study, we focus on the potential role of macrophage-derived cortisol on their polarization in fish. Therefore, we studied the expression of molecules involved in cortisol synthesis and conversion, as well as the expression of cortisol binding receptors in differentially polarized carp macrophages. Moreover, we measured the levels of cortisol and cortisone in M1 and M2 macrophages. Lastly, we analyzed how inhibition of 11β-HSDs and CYP11b, as well as blocking of GRs or PPARγ, affects macrophage polarization/activity.

## 2. Results

### 2.1. Constitutive Expression of Genes Involved in Cortisol Conversion in Lymphoid Organs and Leukocytes

**Figure 1:**
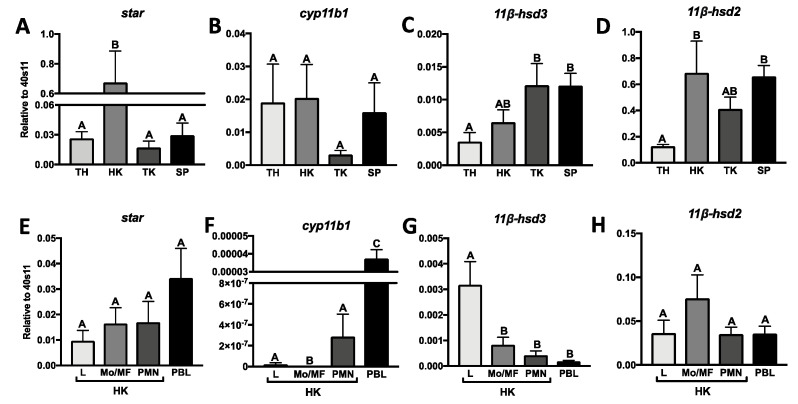
Constitutive expression of genes involved in the (i) synthesis: steroidogenic acute regulatory protein (*star*, (**A**,**E**)), 11β-hydroxylase (*cyp11b*, (**B**,**F**)) and (ii) conversion of cortisol like 11β-hydroxysteroid dehydrogenase type 3 (*11β-hsd3*, (**C**,**G**)) and type 2 (*11β-hsd2*, (**D**,**H**)) in lymphoid organs and the head kidney leukocytes of common carp. Thymus (TH), head kidney (HK), trunk kidney (TK), spleen (SP), peripheral blood leukocytes (PBL), lymphocytes (L), monocytes/macrophages (Mo/MF), neutrophilic granulocytes (PMN). Gene expression was determined by quantitative RT-PCR and expressed relative to expression of the 40S ribosomal protein 11 gene. Data are presented as averages and standard errors (SEs) (*n* = 7–8). Mean values not sharing letters are statistically different.

The lymphoid organs and the leukocyte fractions derived from the head kidney and peripheral blood showed constitutive expression of genes involved in cortisol conversion. The highest expression of *star* was found in the head kidney, while expression of *cyp11b1* was similar in all tested organs. Constitutive expression of *11β-hsd3* and *11β-hsd2* was lowest in the thymus (Figure 1A–D). Within leukocyte populations, the gene expression of star did not differ significantly between cell populations, while the highest constitutive expression of *11β-hsd3* was found in the head kidney lymphocytes. The expression of *11β-hsd2* was comparable in all tested leukocyte populations. The expression of *cyp11b* was exceptionally low in lymphocytes and neutrophilic granulocytes of the head kidney, as well as in peripheral blood leukocytes (PBLs), while it was absent in head kidney monocytes/macrophages (Figure 1E–H).

### 2.2. Expression of Proinflammatory Cytokines/Chemokines and CXC Receptors in Monocytes/Macrophages Stimulated with rcIFN-γ2 + LPS and IL-4/13b

**Figure 2:**
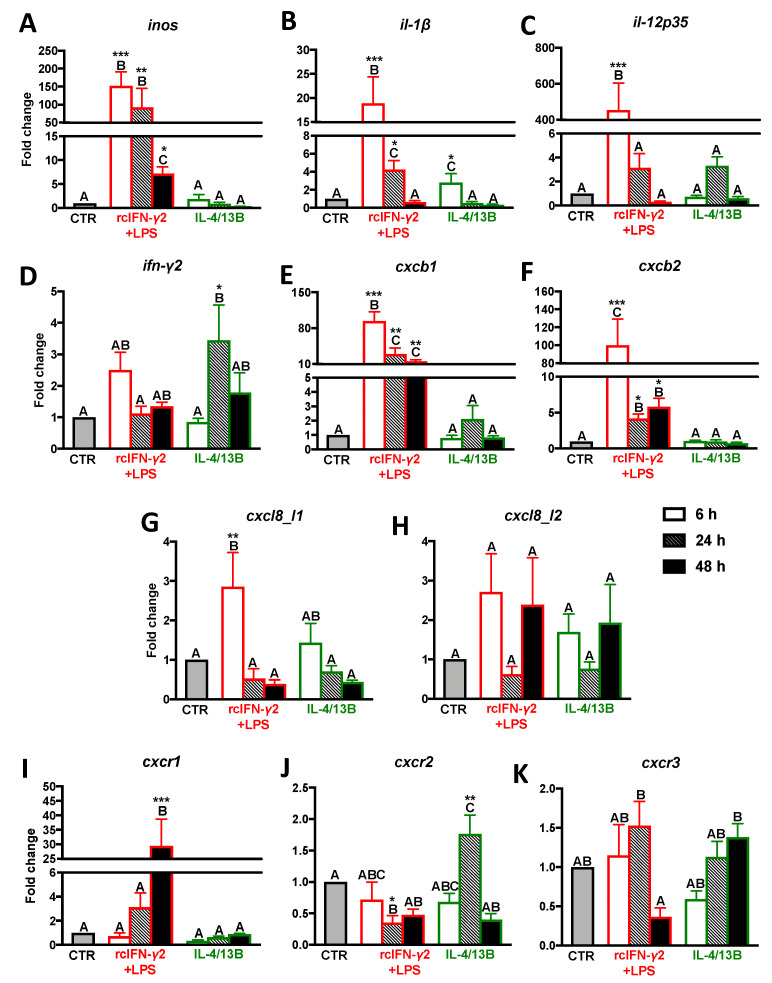
Changes in the expression of proinflammatory molecules (**A**–**D**), CXC chemokines (**E**–**H**), and their receptors (**I**–**K**) in the head kidney monocytes/macrophages. Cells were in vitro treated for 6, 24, or 48 h with lipopolysaccharide (30 μg/mL) combined with recombinant carp interferon gamma2 (100 ng/mL, rcIFN-γ2 + LPS), recombinant interleukin 4/13B (IL-4/13B) (100 ng/mL), or culture medium (CTR). Changes in gene expression are shown as *x*-fold increase compared to unstimulated cells (CTR) and they were standardized for the housekeeping gene 40S ribosomal protein s11. Data are presented as averages and SEs (*n* = 5–6). Mean values not sharing letters are statistically different, while stars (*) indicate statistically significant differences between control (CTR) and treated cells (rcIFN-γ2 + LPS or IL-4/13B) (* *p* ≤ 0.05, ** *p* ≤ 0.001, *** *p* ≤ 0.0001).

After a 6 h treatment with rcIFN-γ2+LPS, monocytes/macrophages showed upregulation of *inos*, *il-1β*, *il-12p35*, *cxcb1*, *cxcb2*, and *cxcl8_l1* gene expression. For *inos*, *cxcb1*, and *cxcb2*, this upregulation was still observed after 24 and 48 h of stimulation, while the upregulation of gene expression of *il-1β* lasted up to 24 h. Moreover, at 48 h of stimulation, rcIFN-γ2 + LPS induced upregulation of *cxcr1* (Figure 2; Table S1).

In monocytes/macrophages treated with IL-4/13B, after 6 h of stimulation, a slight upregulation of expression of the *il-1β* gene was observed, while, after 24 h of IL-4/13B-stimulation, the upregulation of expression of *ifn-γ2* and *cxcr2* was found. At this time point, rcIFN-γ2 + LPS downregulated gene expression of *cxcr2* (Figure 2; Table S1).

### 2.3. Expression of Anti-Inflammatory Mediators in Monocytes/Macrophages Stimulated with IFN-γ2 + LPS and IL-4/13b

**Figure 3:**
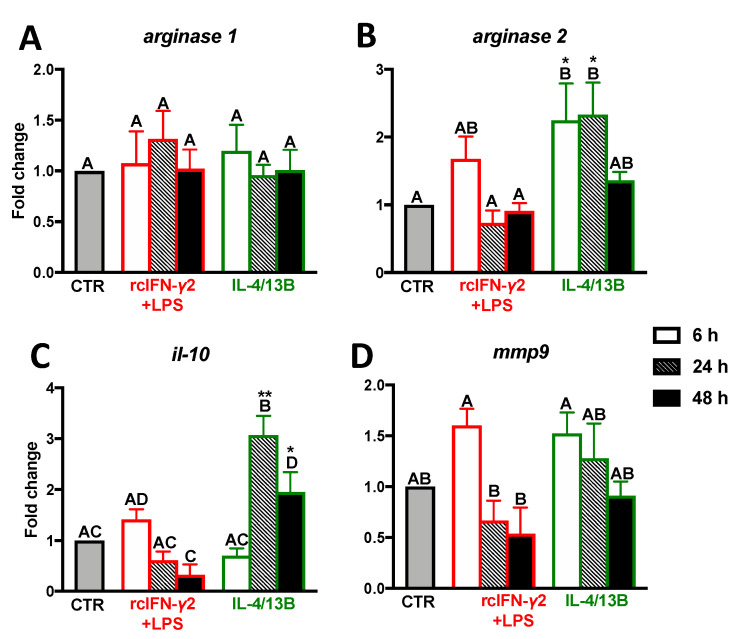
Changes in the expression of anti-inflammatory mediators (**A**–**D**) in the head kidney monocytes/macrophages. Cells were in vitro treated for 6, 24, or 48 h with a combination of lipopolysaccharide (30 μg/mL) and recombinant carp interferon gamma2 (100 ng/mL, rcIFN-γ2 + LPS), recombinant interleukin 4/13B (IL-4/13B) (100 ng/mL), or culture medium (CTR). Changes in gene expression are shown as *x*-fold increase compared to unstimulated cells (CTR) and they were standardized for the housekeeping gene 40S ribosomal protein s11. Data are presented as averages and SEs (*n* = 5–6). Mean values not sharing letters are statistically different, while stars (*) indicate statistically significant differences between control (CTR) and treated cells (rcIFN-γ2 + LPS or IL-4/13B) (* *p* ≤ 0.05, ** *p* ≤ 0.001).

After 6 h of treatment with rcIFN-γ2 + LPS, the gene expression of anti-inflammatory mediators in monocytes/macrophages remained unaffected. In contrast, in monocytes/macrophages treated with IL-4/13B, upregulation of the *arginase 2* gene was found at 6 and 24 h of stimulation. At 24 and 48 h of IL-4/13B-treatment, upregulated expression of *il-10* was also found (Figure 3; Table S1).

### 2.4. Activity of iNOS and Arginase in Monocytes/Macrophages after Stimulation with rcIFN-γ2 + LPS or IL-4/13B

**Figure 4:**
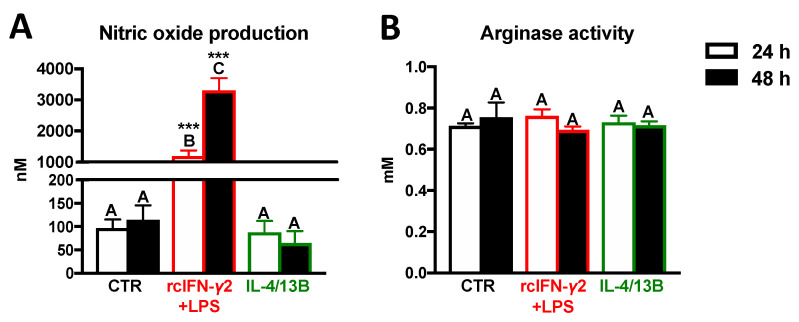
Production of nitric oxide (NO) (**A**) and arginase activity (**B**) in the head kidney monocytes/macrophages. Cells were in vitro treated for 24 or 48 h with a combination of lipopolysaccharide (30 μg/mL) and recombinant carp interferon gamma2 (100 ng/mL, rcIFN-γ2 + LPS), recombinant interleukin 4/13B (IL-4/13B) (100 ng/mL), or culture medium (CTR). Data are presented as averages and SEs (*n* = 5–6). Mean values not sharing letters are statistically different, while stars (*) indicate statistically significant differences between control (CTR) and treated cells (rcIFN-γ2 + LPS or IL-4/13B) (*** *p* ≤ 0.0001).

After 24 and 48 h of rcIFN-γ2 + LPS stimulation, increased levels of NO were found in supernatants from monocyte/macrophage cultures. In contrast, IL-4/13B did not affect NO production. Neither rcIFN-γ2 + LPS nor IL-4/13B influenced the arginase activity (Figure 4).

### 2.5. Expression of Genes Encoding Molecules Involved in Cortisol Conversion and Binding in Monocytes/Macrophages Stimulated with rcIFN-γ2+LPS and IL-4/13B

**Figure 5:**
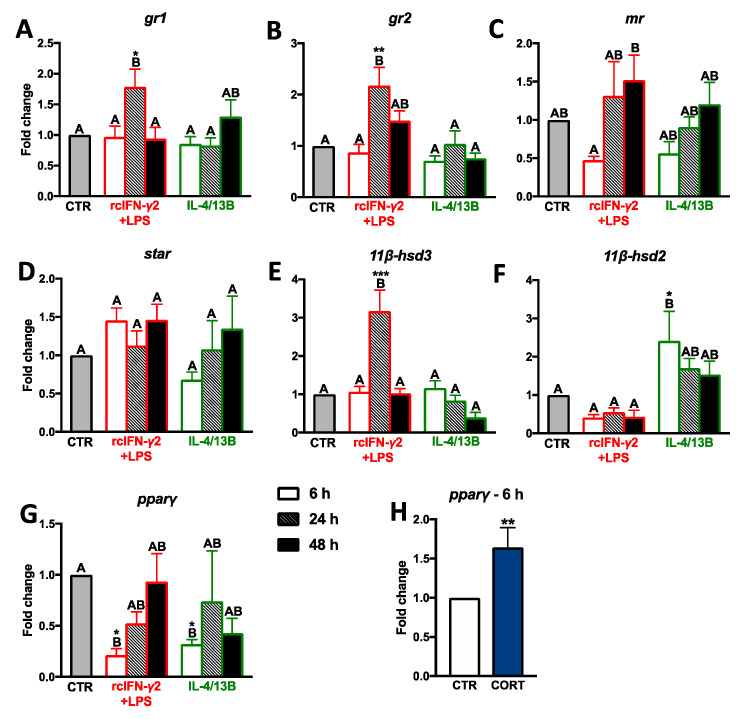
Changes in the expression of glucocorticoid receptors (**A**–**C**), molecules involved in the cortisol synthesis/conversion (**D**–**F**), and peroxisome proliferator-activated receptor gamma (PPARγ) (**G**) in the head kidney monocytes/macrophages. Cells were in vitro treated for 6, 24, or 48 h with a combination of lipopolysaccharide (30 μg/mL) and recombinant carp interferon gamma2 (100 ng/mL, rcIFN-γ2 + LPS), recombinant interleukin 4/13B (IL-4/13B) (100 ng/mL), or culture medium (CTR) or for 6 h with cortisol (CORT, 1 μM) (**H**). Changes in gene expression are shown as *x*-fold increase compared to unstimulated cells (CTR), and they were standardized for the housekeeping gene 40S ribosomal protein s11. Data are presented as averages and SEs (*n* = 5). Mean values not sharing letters are statistically different, while stars (*) indicate statistically significant differences between control (CTR) and treated cells (rcIFN-γ2 + LPS, IL-4/13B or CORT) (* *p* ≤ 0.05, ** *p* ≤ 0.001, *** *p* ≤ 0.0001).

After 24 h of treatment, rcIFN-γ2 + LPS upregulated gene expression of *gr1*, *gr2*, and *11β-hsd3* in monocytes/macrophages. Gene expression of *11β-hsd2* was upregulated at 6 h in cells stimulated with IL-4/13B (Figure 5; Table S1). Moreover, after 6 h of treatment with rcIFN-γ2 + LPS or IL-4/13B, monocytes/macrophages showed downregulation of *pparγ* gene expression (Figure 5G). Additionally, in monocytes/macrophages treated for 6 h with cortisol, upregulation of *pparγ* gene expression was found (Figure 5H).

### 2.6. Cortisone and Cortisol Levels in Monocytes/Macrophages after Stimulation with rcIFN-γ2 + LPS or IL-4/13B

**Figure 6:**
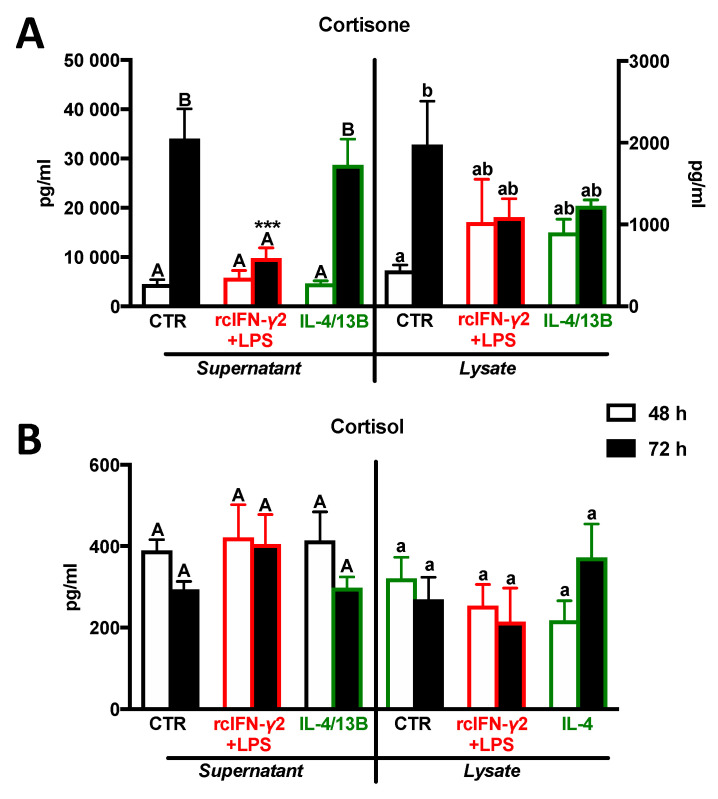
Level of cortisone (**A**) and cortisol (**B**) in head kidney monocyte/macrophage lysates and supernatants. Cells were in vitro treated for 24 h with a combination of lipopolysaccharide (30 μg/mL) and recombinant carp interferon gamma2 (100 ng/mL, rcIFN-γ2 + LPS), recombinant interleukin 4/13B (IL-4/13B) (100 ng/mL), or culture medium (CTR), and, after this time, 15 nM cortisone was added to the cell culture for next 24 or 48 h. Data are presented as averages and SEs (*n* = 5–6). Mean values not sharing letters are statistically different, while stars (*) indicate statistically significant differences between control (CTR) and treated cells (rcIFN-γ2 + LPS or IL-4/13B) at a specific time point (*** *p* ≤ 0.0001).

After 72 h of treatment with rcIFN-γ2 + LPS, supernatants from monocyte/macrophage cultures contained decreased levels of cortisone (Figure 6A). Neither rcIFN-γ2 + LPS nor IL-4/13B affected the cortisol levels in supernatants and lysates from monocyte/macrophage cultures (Figure 6).

### 2.7. The Effect of Cortisol Conversion/Inhibition and GR and PPARγ Blocking on the Monocyte/Macrophage Polarization

**Figure 7:**
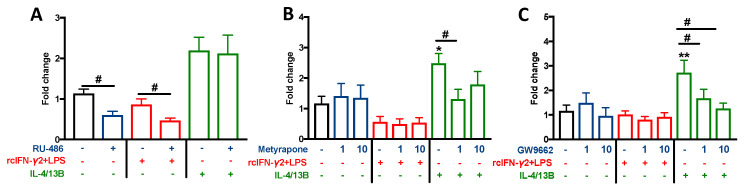
Changes in the gene expression of anti-inflammatory IL-10 in the head kidney monocytes/macrophages. Cells were in vitro pretreated for 1 h with GR antagonist—RU-486 (1 µM) (**A**), inhibitor of cortisol synthesis/conversion—metyrapone (1 µM, 10 µM) (**B**), selective PPARγ antagonist—GW9662 (1 µM, 10 µM) (**C**), or vehicle-dimethyl sulfoxide (DMSO) and then incubated for 48 h with a combination of lipopolysaccharide (30 µg/mL) and recombinant carp interferon gamma2 (100 ng/mL, rcIFN-γ2 + LPS), recombinant interleukin 4/13B (IL-4/13B) (100 ng/mL), or culture medium. Changes in gene expression are shown as *x*-fold increase compared to DMSO- and culture medium-treated cells, and they were standardized for the housekeeping gene 40S ribosomal protein s11. Data are presented as averages and SEs (*n* = 5–6). Stars indicate significant differences between control and stimulated cells (* *p* ≤ 0.05, ** *p* ≤ 0.01). Number signs indicate significant differences between control cells and cells treated with GR antagonist (# *p* ≤ 0.05).

All studied inhibitors/antagonists affected gene expression of IL-10 in carp monocytes/macrophages. In unstimulated and rcIFN-γ2 + LPS-treated cells, RU-486 downregulated its expression, while, in IL-4/13B-treated cells, RU-486 did not change *il-10* expression (Figure 7A). Both metyrapone and GW9662 downregulated IL-4/13B-induced expression of *il-10* (Figure 7B,C).

Moreover, unstimulated monocytes/macrophages, treated with RU-486, showed downregulation of *inos* and *il-12p35* expression and upregulation of *cxcr1* gene expression. In rcIFN-γ2 + LPS-treated cells, RU-486 upregulated the expression of *inos* and *cxcr1*, while RU-486 upregulated *cxcr1*, *cxcr3*, and *pparγ* expression in IL-4/13B-stimulated cells (Figure S1). RU-486, metyrapone, and GW9662 did not alter NO production or arginase activity in M1 and M2 cells (data not shown).

In unstimulated monocytes/macrophages, metyrapone upregulated the expression of *inos* and *cxcr1* and downregulated the expression of *cxcr3*. Metyrapone also reversed the IL-4/13B-induced upregulation of *arginase 2*, while it slightly increased *inos* expression (Figure S2). In rcIFN-γ2 + LPS-treated cells, the PPARγ antagonist downregulated expression of *cxcr2* (Figure S3). The PPARγ antagonist alone upregulated gene expression of *gr1*, *star*, and *11β-hsd2* (Figure S4). It also increased star expression in both M1 and M2 macrophages, while, in M1 macrophages, upregulation of *11β-hsd3* expression upon GW9662 treatment was found. In turn, in IL-4/13B-stimulted cells, it increased *gr1* and *2* expression (Figure S4), while it did not affect the *cyp11b1* expression (data not shown).

## 3. Discussion

This in vitro study focused on the role of macrophage-derived cortisol on cell polarization toward inflammatory versus regenerative functions. We found that, in carp, both lymphoid organs and leukocyte populations constitutively express genes that are required for cortisol synthesis/conversion (*star*, *cyp11b*, *11β-hsd3* and *2*). As expected, the highest expression of these genes was observed in the head kidney, which, in teleost fish, in addition to its hematopoietic and immunocompetent functions, is the equivalent of the mammalian adrenal gland and produces cortisol [6]. Moreover, our studies indicated that cortisol can also be produced and converted in the other lymphoid organs and in leukocytes, predominantly in monocytes/macrophages. The extra-adrenal production/conversion of cortisol/corticosterone has been reported previously in mammalian and in avian lymphoid organs [33,34,35]. For example, murine epithelial cells of thymus and thymocytes showed expression of genes encoding pivotal molecules involved in steroidogenesis including *star*, *cyp11a*, and *cyp11b1* [36]. It was, therefore, hypothesized that the presence of steroidogenic enzymes in central lymphoid organs is probably critical for lymphocyte differentiation and selection [37,38]. Furthermore, expression of these genes was discovered in different leukocyte populations of rodents and humans [39,40] and in monocyte/macrophage cell lines (RAW264.7 and THP-1) [14,41]. Expression of star and *cyp11b* was also confirmed in fish, both in steroidogenic and in non-steroidogenic tissues. Not surprisingly, *star* and *cyp11b* expression was found mainly in the head kidney (for a review, see Tokarz and coworkers [10]), but was additionally manifested in the intestine, spleen, and trunk kidney [42].

Although, in mammals, 11β-HSD2 is mainly expressed in aldosterone-target tissues [8], it was also found in thymus, bone marrow, and spleen [43]. Intriguingly, to our knowledge, 11β-HSD2 expression has not been demonstrated in human and mice white blood cells, except during conditions of disease like rheumatoid arthritis or acute respiratory distress syndrome [7,44,45,46,47].

As in mammals, the main function of 11β-HSD2 in fish is the conversion of cortisol to cortisone in order to avoid excessive activation of intracellular receptors (GRs and MRs) upon high cortisol levels [48,49]. Therefore, it is mainly found in the brain, gills, liver, heart, spleen, intestine, muscle, head kidney, and gonads [20,48,49,50,51]. So far, its expression has not been studied in fish leukocytes.

In turn, in mammals, 11β-HSD1, which reduces cortisone to the active cortisol, is widely expressed in the liver, adipose tissue, muscle, pancreatic islets, adult brain, and gonads, as well as in lymphoid organs [52,53]. Moreover, 11β-HSD1 expression was observed in mammalian macrophages, neutrophils, mast cells, and dendritic cells [7,46,54,55,56], as well as in T- and B-cells [57]. Surprisingly, phylogenetic analysis showed that teleost fish do not possess a direct homolog to 11β-HSD1 [19,20]. Instead they express its paralog and most probably its ancestor 11β-HSD3, known also as 11β-HSD1-like protein [19]. This 11β-HSD isoform was previously described in mammals as an enzyme oxidizing cortisol to cortisone, but not the reverse reaction, albeit at high substrate concentrations and indirectly measured [58]. In contrast to humans, zebrafish have two *11β-hsd3* genes (*11β-hsd3a* and *11β-hsd3b*) and both have been described as enzymes with 11-oxidoreductase activity [19,20,58]. In turn, Tsachaki and coauthors [59] found that, in zebrafish, neither human 11β-HSD3 nor the two zebrafish 11β-HSD3 homologs converted cortisone and 11-ketotestosterone (11KT) to their 11β-hydroxyl forms. Therefore, until now, the function of this enzyme was not fully clear. In zebrafish, *11β-hsd3* expression was confirmed in fish liver, skin, gonads, eyes, muscle, heart, kidney, spleen, intestine, and gills [19,20,60], but it was not studied in leukocytes.

In the next step, we verified the time-dependent changes in the expression of M1 and M2 markers in monocytes/macrophages stimulated with rcIFN-γ2 + LPS or IL-4/13B. We found that, in these cells, rcIFN-γ2 + LPS stimulation caused fast (6 h) upregulation of pro-inflammatory mediators and that this reaction was time-dependent. In contrast, IL-4/13B stimulation upregulated anti-inflammatory *arginase 2* and *il-10* expression. A strong proinflammatory stimulation was previously described in carp leukocytes treated with IFN-γ + LPS. This was manifested by upregulation of expression of *inos*, *il-12p35*, and the chemokines *cxcl8_l2* and *cxcb* and by an increased production of NO [61,62]. Correspondingly, in head kidney and peritoneal leukocytes of Asian sea bass, stimulation with IFN-γ + LPS increased the production of NO [63], while, in head kidney macrophages of grass carp, IFN-γ + LPS upregulated the expression of proinflammatory mediators (*il-1β*, *il-6*, *inos*, *tnf-α*) and *mhcII* [64].

In turn, in IL-4/13-treated fish leukocytes, a reduced production/expression of proinflammatory mediators was found. Moreover, in these cells, IL-4/13 increased the arginase activity and the expression of anti-inflammatory cytokines e.g., *il-10* and *tgf-β* [24,26,65].

Considering our present results and data mentioned above, we can conclude that stimulation of carp monocytes/macrophages with rcIFN-γ2 + LPS and with IL-4/13B induces M1 and M2 polarization, respectively.

In these polarized monocytes/macrophages, we measured gene expression and activity of enzymes involved in cortisol conversion, as well as gene expression of GR, MR, and PPARγ. We found that 24 h of rcIFN-γ2 + LPS stimulation upregulated the expression of genes encoding GRs as well as *11β-hsd3*, while IL-4/13B stimulation upregulated the expression of *11β-hsd2* (at 6 h of stimulation). Moreover, in supernatants from rcIFN-γ2 + LPS-treated cells, a decreased level of cortisone was found. This may suggest that, upon stimulation, monocytes/macrophages more actively convert this hormone; however, no differences in cortisol level were observed at 48 and 72 h of culture.

Earlier, indications were found that expression/activity of 11*β*-HSDs alters upon macrophage polarization [7,44,66,67]. For example, stimulation of human macrophages with LPS + IFN-γ induced a higher *11β-hsd1* gene expression than stimulation with IL-4 [68]. In contrast, Freeman and coauthors [46] found increased 11β-HSD1 activity upon granulocyte-macrophage colony-stimulating factor (GM-CSF)- or IL-4-induced differentiation of monocytes into immature dendritic cells. Similar results were also obtained by Thieringer and colleagues [7] who demonstrated that *11β-hsd1* is expressed only upon differentiation of human monocytes to macrophages, while *11β-hsd2* was detected neither in monocytes nor in cultured human macrophages. Moreover, they found that, compared to resting macrophages, the 11β-HSD1 level is high in proinflammatory cells but even higher in IL-4- or IL-13-stimulated anti-inflammatory macrophages. Furthermore, it was observed that incubation with LPS increased the expression of *11β-hsd1* in proinflammatory macrophages, but not in monocytes [7]. An interesting hypothesis was put forward by Chapman and coworkers [8], who suggested that high expression of *11β-hsd1* in M1 macrophages may promote their subsequent transition to a M2 phenotype to attain clearing of debris and apoptotic cells and, consequently, to promote resolution of inflammation. Similarly, differentiation of monocytes in the presence of glucocorticoid generates a highly phagocytic macrophage phenotype while glucocorticoid treatment of macrophages promotes phagocytosis [52]. These observations underline the important role of glucocorticoids in accelerating the phagocytosis of apoptotic cells by macrophages, in order to terminate the inflammation and to start the repair and wound healing phase.

Changes in HSD expression/activity were also observed in leukocytes during chronic inflammatory diseases. For example, in rats with colitis, an increased expression of *11β-hsd1* was found in the mesenteric lymph nodes and in lymphoid cells [69], while, in synovial macrophages of patients with osteoarthritis or rheumatoid arthritis, increased expression of *11β-hsd2* and *11β-hsd1* was observed [44,70]. Moreover, a high level of *11β-hsd2* was found in alveolar macrophages of patients with acute respiratory distress syndrome [71].

We now could demonstrate changes in 11β-HSD expression in fish leukocytes after rcIFN-γ2 + LPS- and IL-4/13B stimulation. This corroborates the earlier observed LPS-induced upregulation of expression of *gr1* encoding genes [72]. Moreover, we found that, in carp peritoneal leukocytes and in the head kidney, *gr1* and *2* gene expression increased during zymosan-induced peritonitis [73].

Furthermore, in mammalian and mice macrophages, an increased expression of *gr* was found after LPS or IFN-γ stimulation [74,75,76,77]. Interestingly, stimulation of M2 macrophages with cortisone caused upregulation of expression of *11β-hsd1* and *pparγ* and downregulation of *il-6*, *tnf-α*, and *il-1β* [66].

To finally clarify the role of macrophage-derived cortisol in polarization of these cells, we used metyrapone, an inhibitor of cortisol synthesis/conversion, and the GR blocker RU-486. Treatment with these inhibitors induced prominent changes in the expression of *il-10*. We found that RU-486 downregulated expression of *il-10* in unstimulated and in rcIFN-γ2 + LPS-stimulted cells, while metyrapone downregulated *il-10* expression in IL-4/13B-stimulated cells. Moreover, in rcIFN-γ2 + LPS-stimulated cells, metyrapone increased the expression of pro-inflammatory *inos* and decreased gene expression of the second M2 marker—*arginase 2*. Together with the upregulation of GR expression, which was found after stimulation with rcIFN-γ2 + LPS, these data suggest that *il-10* expression depends on the availability of intracellular cortisol, and that intracrine interaction of endogenous cortisol with intracellular GRs stimulates the anti-inflammatory response in macrophages, in order to prevent excessive proinflammatory activation which could damage healthy host cells. This hypothesis also corroborates that, with the time-dependent changes in macrophage activation, high expression of proinflammatory mediators preceded upregulation of the gene expression of *11β-hsd3* and GRs. Furthermore, these data indicate that inhibition of cortisol synthesis/conversion decreased IL-4/13B-stimulated M2 macrophage polarization.

Previously, RU-486-induced decrease of IL-10 was observed e.g., in human M2 macrophages, while RU-486 could not change the activity of M1 macrophages [78]. Furthermore, experiments with *11β-HSD1^−^/^−^* mice and experiments with cells/animals treated with different 11β-HSD1 blockers revealed an immunoregulatory role for the 11β-HSD1 enzyme expressed in leukocytes specifically in regulation of macrophage activity [44].

For example, Zhang and Daynes [56] found that, in vivo, *11β-HSD1^−^/^−^* mice are more susceptible to LPS-induced endotoxemia and that their peritoneal and splenic macrophages overproduce proinflammatory cytokines following LPS stimulation in vitro. The latter phenomenon was associated with an increased activation of the nuclear factor kappa-light-chain-enhancer of activated B cells (NF-κB) and mitogen-activated protein kinase (MAPK) signaling cascades. Moreover, it was shown that macrophages of *11β-HSD1^−^/^−^* mice show lower/delayed phagocytic activity against apoptotic neutrophils [47]. Interestingly, these results were confirmed by in vitro studies of murine macrophages, where 11β-HSD1 inhibitor abolished the 11-dehydrocorticosterone-stimulated phagocytic activity [47]. Moreover, the use of an 11β-HSD1 inhibitor impaired the resolution of inflammation and promoted angiogenesis in murine models of surgical wound healing. This indicates that this enzyme is relevant for tissue remodeling after inflammation [35,79]. Such effects were also observed upon metyrapone treatment, which resulted in accelerated wound healing of human skin ex vivo and in vivo [80]. Moreover, in mice with colitis, metyrapone inhibited TNF-α-induced glucocorticoid synthesis [81]. These results indicate anti-inflammatory properties of this enzyme and a potential function in macrophage polarization. In contrast, Ishii and coworkers [67] found that inhibition of 11β-HSD1 in LPS-treated J774.1 macrophages suppressed the expression and secretion of proinflammatory cytokines. Similar results were obtained in vivo in mice [82].

Taking into account the anti-inflammatory action of PPARγ in mammalian macrophages [5,83,84] and the fact that PPARγ was shown to activate *11β-hsd1* expression in macrophages [66], we measured changes in *pparγ* expression in carp M1 and M2 cells. Furthermore, we determined if and how a selective PPARγ antagonist (GW9662) would affect the expression of the polarization markers. We found that a short-term stimulation (6 h) with rcIFN-γ2 + LPS and, surprisingly, stimulation with IL-4/13B downregulated the expression of *pparγ*, while cortisol upregulated its expression.

Moreover, PPARγ blocking reduced *il-10* expression in IL-4/13B-treated monocytes/macrophages. The latter observation confirms previous reports stating that pharmacological activation of PPARγ attenuated the proinflammatory response in macrophages [84,85,86,87,88]. For example, Bouhlel and coworkers [85] found that a lack of PPARs led to impairment of M2 macrophage function, while the PPARγ agonist abolished the LPS- or IFN-γ-induced changes in gene expression in murine macrophages [84]. Similar observations were also described for fish. For example, overexpression of PPARγ in spleen cells of the orange-spotted grouper decreased the expression of proinflammatory mediators, whereas blocking of PPARγ induced an increase in inflammation [89]. Interestingly, in human macrophages, as with dexamethasone, GW9662 shifted the phenotype of macrophages from M2a to M2c, characterized by an inhibited production of TNF-α and IL-10 [90].

We, therefore, conclude that carp monocytes/macrophages can convert cortisol from and to its inactive form cortisone. This endogenous cortisol, via direct interaction with intracellular GRs in macrophages (intracrine interaction), is most probably required for the IL-10-dependent control of their proinflammatory activity and for the regulation of macrophage polarization. These data suggest that changes in the expression of 11β-HSDs and most probably in their activity in M1 and M2 macrophages may contribute to their regulatory function during the immune response, with potential consequences for inflammatory diseases. Finally, our findings demonstrate evolutionarily conserved crosstalk among the nuclear receptors PPARγ, GR, and inflammatory molecules.

Despite extensive in vitro studies, a full understanding of the physiological importance of the endogenous cortisol metabolism in macrophages will require in vivo verification with cell-specific knockout of genes encoding GRs and/or cortisol-converting enzymes in macrophages.

## 4. Materials and Methods

### 4.1. Animals

Juvenile common carp (*Cyprinus carpio* L., body weight 60–90 g, 9–12 months R3xR8) were obtained from the Institute of Ichthyobiology and Aquaculture, Polish Academy of Science, Golysz, Poland. After the transport, animals were adjusted for 4 weeks at 21 °C and a 12 h/12 h light/dark cycle in recirculating tap water at the Institute of Zoology and Biomedical Research in Krakow, Poland. Fish were kept in tanks (volume 375 L, flow rate 4 L/min, density 45 fish/tank and 60 g/L) and fed pelleted dry food (Aller Master, Aller Aqua, Czarna Dabrowka, Poland) at a daily maintenance rate of 1% of their estimated body weight. In order to avoid additional stress and/or differences in handling, all samplings were performed by the same person and at the same time of day (at 9.00 a.m.). All animals were handled in strict accordance with good animal practice as defined by the relevant national and local animal welfare bodies, and procedures were approved by the local ethical committee (2nd Local Institutional Animal Care and Use Committee (IACUC) in Krakow, Poland (license number 292/2017).

### 4.2. Isolation of Lymphoid Organs, Peripheral Blood Leukocytes, and Head Kidney Leukocytes

Animals were anaesthetized with tricaine methane sulfonate (TMS; Sigma-Aldrich, St. Louis, MO, USA; 0.2 g/L) buffered with NaHCO_3_ (POCH, Gliwice, Poland; 0.4 g/L), and blood from the tail vein was collected with a needle attached to a 5 mL syringe with carp RPMI (cRPMI 1640, Lonza, Walkersville, MD, USA, adjusted to carp osmolarity of 270 mOsm/kg^−1^ with distilled water) containing 0.066 mg/mL heparin (Sigma-Aldrich, St. Louis, MO, USA). The blood was centrifuged for 10 min at 800× *g* at 4 °C, and the buffy coat was layered on Histopaque (Sigma-Aldrich, St. Louis, MO, USA) to isolate the pure population of peripheral blood leukocytes (PBLs) and centrifuged for 25 min at 800× *g* at 4 °C with the brake disengaged. Cells were collected and washed two times in cRPMI; then, the cell pellet was resuspended in to RL buffer (Eurx, Gdansk, Poland) with 1% β2-mercaptethanol and kept at −80 °C for gene expression analyses.

Thymus, head kidney, trunk kidney, and spleen were carefully removed, transferred to fix RNA buffer (Eurx, Gdansk, Poland), and kept at −80 °C for gene expression analyses.

Head kidney cell suspensions were obtained by passing the organ through 100 µm nylon mesh (BD Biosciences, Bedford, MA, USA) with cRPMI containing 0.051 mg/mL heparin (Sigma-Aldrich, St. Louis, MO, USA) and washed once for 10 min at 800× *g*. The cell suspension was layered on a discontinuous Percoll gradient (1.020, 1.060, 1.070, and 1.083 g/cm^3^) (GE Healthcare, Uppsala, Sweden) and centrifuged for 30 min at 800× *g* with the brake disengaged. The cell layers (1.020 g/cm^3^ (lymphocytes); 1.060 g/cm^3^ (monocytes/macrophages); 1.07 g/cm^3^ (granulocytes (PMN)) were collected and washed two times in cRPMI (10 min, 800× *g*, 4 °C) [91]. Composition/purity of leukocyte population was analyzed as described previously [32]. Briefly, cells were stained with neutral red (NR, 0.1 mg/mL, 3 min, at RT (Sigma-Aldrich, USA)) or with Türk’s solution (0.01% crystal violet (Sigma-Aldrich, USA) in 3% acetic acid (Chempur, Piekary Slaskie, Poland), 3 min, at RT) and analyzed in a hemocytometer. Moreover, cell size (FSC) and granularity (SSC) were measured with a FACScalibur flow cytometer (BD Biosciences). Data were analyzed using WinMDI 2.9 software (Joe Trotter, http://facs.scripps.edu). Additionally, the constitutive gene expression of markers for T-lymphocytes (*lck*), B-lymphocytes (*IgM heavy chain*), monocytes/macrophages (*csf1r*), and granulocytes (*mpx*) was measured by RQ-PCR (Figure S5).

### 4.3. Monocytes/Macrophages Culture and Polarization

To induce cell polarization, the enriched monocyte/macrophage suspension, containing 68.8% ± 1.05% monocytes/macrophages, 4.02% ± 0.41% lymphocytes, and 27.18% ± 1.15% PMNs, was used. A detailed characterization of this cell population was described previously [32,92].

The enriched monocyte/macrophage suspension was resuspended to a density of 10 million cells per 1 mL in cRPMI++ (cRPMI supplemented with 0.5% (*v*/*v*) pooled carp serum and antibiotics (1% l-glutamine (Sigma-Aldrich, St Louis, MO, USA), 1% (*v*/*v*) penicillin G (Sigma-Aldrich, St Louis, MO, USA), and 1% (*v*/*v*) streptomycin sulfate (Sigma-Aldrich, St Louis, MO, USA)). Cells were seeded either in 96-well cell culture plates (Nest Biotech Co., Wuxi, China) to measure arginase and iNOS activity or in 24-well cell culture plates (Nest Biotech Co., China) to measure gene expression. Cells were treated with lipopolysaccharide (LPS, *Escherichia coli* serotype O55: B5, 30 µg/mL, Sigma-Aldrich, St. Louis, MO, USA [61]) in combination with recombinant carp interferon gamma2 (rcIFN-γ2, 100 ng/mL) or with zebrafish (*Danio rerio*) recombinant IL-4/13B (100 ng/mL, Kingfisher Biotech. Inc., St. Paul, MN, USA [26]) for 6, 24, and 48 h at 27 °C in 5% CO_2_ atmosphere. Additionally, monocytes/macrophages were treated for 6 h with cortisol (Sigma–Aldrich, St. Louis, MO, USA, 1 μM). Sequence alignments of carp (BAM09149.1 and BAL43179.1) and zebrafish (CAL48253.2) IL-4/13B were made with ClustalW within the MEGA4 software (Figure S6).

### 4.4. Inhibition of Cortisol Conversion and Blocking of GR and PPARγ

To verify involvement of macrophage-derived cortisol in macrophage polarization, cells were pretreated for 1 h with an inhibitor of cortisol synthesis/conversion—metyrapone (Sigma-Aldrich, St. Louis, MO, USA; 1 µM, 10 µM), while, to study role of GRs and PPARγ in this process, cells were pretreated with GR antagonist—RU-486 (Sigma-Aldrich, St. Louis, MO; 1 µM [93]) or selective PPARγ antagonist—GW9662 (Sigma-Aldrich, St. Louis, MO; 1 µM, 10 µM [89]). All inhibitors were dissolved in dimethyl sulfoxide (DMSO) (Bioshop, Burlington, ON, Canada); therefore, control cells were pretreated with vehicle—0.1%, DMSO. After 1 h of pretreatment cells were stimulated for 48 h with LPS + IFN-γ2 or IL-4/13B as described in Section 2.3, there were no changes in cell survival after treatment with stimulators and/or inhibitors as measured with PrestoBlue test (Invitrogen Corporation, San Diego, CA, USA).

### 4.5. Arginase Activity Assay

Arginase activity was measured as described by Corraliza and coworkers [94]. Cells were lysed in 50 µL of 0.1% Triton X-100 (Biorad, Hercules, CA, USA) containing 5 µg of antipain dihydrochloride from microbial source, 5 µg of aprotinin from the bovine lung, and 5 µg of pepstatin A (all from Sigma-Aldrich, St. Louis, MO, USA) at room temperature for 30 min on a shaker. Next, 35 µL of 10 mM MnCl_2_ (Sigma-Aldrich, St. Louis, MO, USA) and 50 mM Tris–HCl (pH 7.5) (Tris—Biorad, Hercules, CA, USA; HCl—POCH, Gliwice, Poland) were added, and the mixture was incubated for 20 min at 55 °C. Next, 50 µL of this activated lysate was transferred to a new tube and 50 µL of 0.5 M l-arginine was added (Sigma-Aldrich, St. Louis, MO, USA) (pH 9.7). The suspension was incubated for 1 h at 37 °C, and the reaction was stopped by adding 400 µL of acid mixture, containing 1 mL of H_2_SO_4_ (96%, POCH, Gliwice, Poland), 3 mL of H_3_PO_4_ (85%, Chempur, Piekary Slaskie, Poland), and 7 mL of H_2_O. In the next step, 25 µL of 9% α-isonitrosopropiophenone (Sigma-Aldrich, St. Louis, MO, USA) in 100% ethanol (POCH, Gliwice, Poland) was added to each sample, and they were subsequently incubated for 45 min at 100 °C. After 10 min of cooling in the dark, the optical density (OD) was read at 540 nm, and the arginase activity was calculated using a urea standard curve (0–6.66 mM).

### 4.6. Nitric Oxide Assay

Nitrite/nitrate production was measured in cell culture supernatants as described previously [95,96]. Following cell incubation, 50 µL of cell culture supernatant was added to 25 µL 1% (*w*/*v*) sulfanilamide in 2.5% (*v*/*v*) phosphoric acid and 25 µL of 0.1% (*w*/*v*) *N*-naphthyl-ethylenediamine in 2.5% (*v*/*v*) phosphoric acid (all from Sigma–Aldrich, St. Louis, MO, USA). The OD was read at 540 nm. The nitrite concentration was calculated with a sodium nitrite standard curve (0–10 µM).

### 4.7. Enzyme Immunoassays

To measure the activity of 11β-HSD3 and 11β-HSD2 in cell cultures, head kidney monocytes/macrophages were resuspended to a density of 5 × 10^6^ cells/mL in carp RPMI++ without 0.5% carp serum and seeded in a 24-well cell culture plate (Nest Biotech Co., China) at 27 °C, 5% CO_2_. Cells were treated as described in Section 2.3 and Section 2.4 and incubated 24 h. After this time, 15 nM cortisone (Sigma-Aldrich, St. Louis, MO, USA) was added to the cell culture. After incubation for 48 or 72 h, supernatants were collected while the cells were suspended in 150 µL of PathScan^®^ Sandwich ELISA Lysis Buffer (Cell Signaling Technology, Leiden, The Netherlands). The cell lysates and supernatants were kept at −20°C for future analysis of cortisone and cortisol levels.

On the day of the assay, the samples were thawed, and hormone levels were determined with commercial kits (for cortisone: Arbor Assays, Ann Arbor, MI, USA) (for cortisol: Neogen, Lexington, KY, USA): (i) for cortisone sensitivity, 29.0 pg/mL; range: 60–100 000 pg/mL, cross reactivity with cortisol <0.1%; (ii) for cortisol sensitivity, 0.04 ng/mL; range: 0.04–10 ng/mL, cross reactivity with cortisone 15.7%.

Levels of cortisol and cortisone were also measured in intact culture medium (without cells) and in control unstimulated cells/supernatants which were not supplemented with cortisone (at 48 h of incubation). In the intact culture medium, very low levels of hormones were found (83.33 ± 15.37 pg/mL of cortisol and 20 ± 16.33 pg/mL of cortisone). Lysates from control cells without stimulation contained 56.67 ± 14.24 pg/mL of cortisol, and, in supernatants from control cells, 220 ± 51.23 pg/mL of cortisol was found. No cortisone was detected in lysate and supernatants from control unstimulated cells.

### 4.8. Gene Expression

#### 4.8.1. Isolation of RNA and Complementary DNA (cDNA) Synthesis

RNA from organs and cells was isolated with a GeneMATRIX Universal RNA Purification Kit (Eurx, Gdansk, Poland) following the manufacturer’s protocol. Final elution was carried out in 30 µL of nuclease-free water, to maximize the concentration of RNA. RNA concentrations were measured by Tecan Spark NanoQuant PlateTM and samples were kept at −80 °C. The cDNA synthesis reaction was performed using a High-Capacity cDNA Reverse Transcription Kits (Applied Biosystems, Waltham, MA, USA) according to the manufacturer’s protocol. Briefly, 1 µg of total RNA was used in the reaction and the final volume was brought to 100 µL with nuclease free-water and stored at −20 °C.

#### 4.8.2. RQ-PCR

All RT-qPCR reactions were performed with a Rotor-Gene Q machine (Qiagen, Hilden, Germany). The total reaction volume included 7 µL of SYBR^®^Select Master Mix (Applied Biosystems, Waltham, MA, USA), 2 µL of forward and reverse primers (Table S2), and 4 µL of the cDNA sample. In all cases, amplification was specific, and no amplification was observed in negative control samples (non-template control, NTC, samples containing nuclease-free water instead of cDNA) and non-reverse transcriptase control (−RT, samples not containing reverse transcriptase). The 40S ribosomal protein s11 gene served as a housekeeping gene. The RQ-PCR program was based on the program for SYBR^®^Select Master Mix (50 °C at 2 min; 95 °C at 2 min, 40 cycles of −95 °C at 15 s, 60 °C at 60 s). To analyze the purity of the PCR products and the specificity of the reaction, following each run, melt curves were made by detecting fluorescence from 60 to 95 °C at 0.5 °C intervals.

Constitutive gene expression was measured as a ratio of target gene vs. reference gene (40S ribosomal protein s11 gene) and calculated with the Pffafl method [97], according to the following equation:

(1) $$\mathrm{Ratio}=\frac{{\left(E_{\mathrm{reference}}\right)}^{{\mathrm{Ct}}_{\mathrm{reference}}}}{{\left(E_{\mathrm{target}}\right)}^{{\mathrm{Ct}}_{\mathrm{reference}}}}$$

where E is the amplification efficiency, and Ct is the number of PCR cycles needed for the signal to exceed a predetermined threshold value.

Gene expression following stimulation was rendered as a ratio of target gene vs. reference gene (40S ribosomal protein s11 gene) relative to the expression in unstimulated control samples according to the following equation:

(2) $$\mathrm{Ratio}=\frac{{\left(E_{\mathrm{target}}\right)}^{∆\mathrm{Ct}}{\mathrm{Target}}^{\left(\mathrm{control}-\mathrm{sample}\right)}}{{\left(E_{\mathrm{reference}}\right)}^{∆\mathrm{Ct}}{\mathrm{Reference}}^{\left(\mathrm{control}-\mathrm{sample}\right)}}$$

### 4.9. Statistical Analysis

Statistical analysis was performed using GraphPad 7 (GraphPad Software, San Diego, CA, USA). Data were expressed as the mean and standard error (SE). The differences between changes in gene expression/cell activity after 6, 24, and 48 h of incubation were compared by a two-way analysis of variance (ANOVA), followed by post hoc Tukey’s test. Bartlett’s test was performed to ensure the suitability of the data for parametric significance tests. Significant differences between changes in gene expression/cells activity in 48 h and constitutive gene expression were performed by one-way ANOVA followed by post hoc Dunnett’s test in the case of normally distributed data or with the nonparametric Kruskal–Wallis test followed by Dunn’s test for data that were not normally distributed. A Shapiro–Wilk test was performed to ensure the suitability of the data for parametric significance tests. Differences in gene expression after 48 h in cells incubated with or without inhibitors were compared using the Mann–Whitney U test for data that were not normally distributed or the *t*-test for normally distributed data. Differences were considered statistically significant at *p* ≤ 0.05

## Supplementary Materials

The following are available online at https://www.mdpi.com/1422-0067/21/23/8954/s1.
