# Supplementary material for: Cortisol Metabolism in Carp Macrophages: A Role for Macrophage-Derived Cortisol in M1/M2 Polarization
Source: Int J Mol Sci. 2020 Nov 25;21(23):8954. doi: 10.3390/ijms21238954 (PMC7728068; doi:10.3390/ijms21238954)
Supplement: Supplementary file 1 [file ijms-21-08954-s001.pdf]

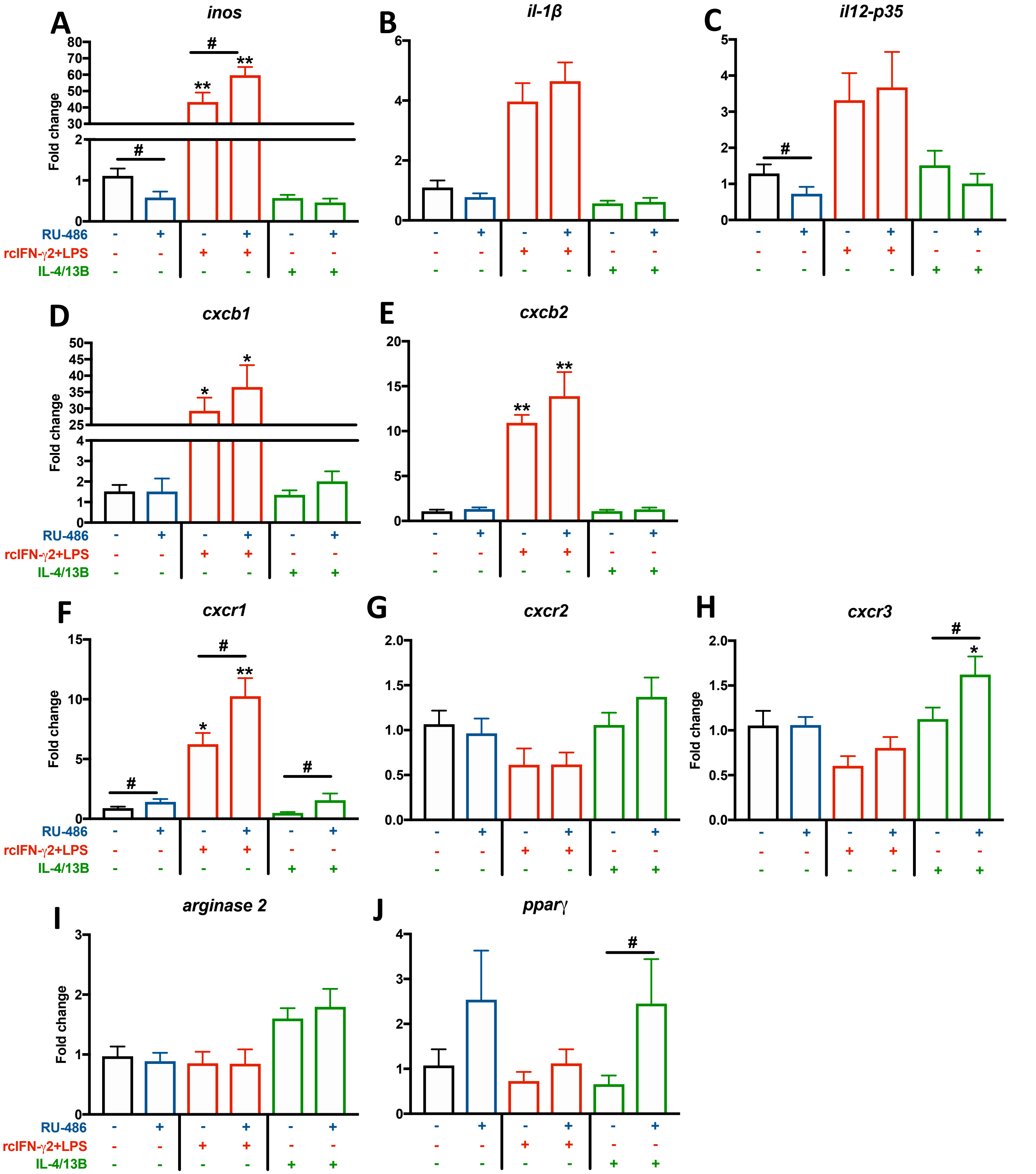

Supplementary Figure 1. Changes in the expression of pro-inflammatory molecules (**A-E**), CXC chemokine receptors (**F-H**), arginase 2 (**I**) and peroxisome proliferator-activated receptor gamma (PPARγ) (**J**) in the head kidney monocytes/macrophages. Cells were *in vitro* pretreated for 1 h with GR antagonist – RU-486 (1 μM) or vehicle (DMSO) and next for 48 h incubated with a combination of lipopolysaccharide (30 μg/mL) and recombinant carp interferon gamma2 (100 ng/mL, rcIFN-γ2+LPS), recombinant IL-4 (100 ng/mL) or culture medium. Changes in gene expression are shown as x-fold increase compared to DMSO- and culture medium-treated cells and they were standardized for the housekeeping gene 40S ribosomal protein s11. Averages and S.E (n=5-6). Stars indicate significant differences between control and stimulated cells (*$p \leq 0.05$, ** $p \leq 0.01$). Number signs indicate significant differences between control cells and cells treated with GR antagonist (#$p \leq 0.05$).

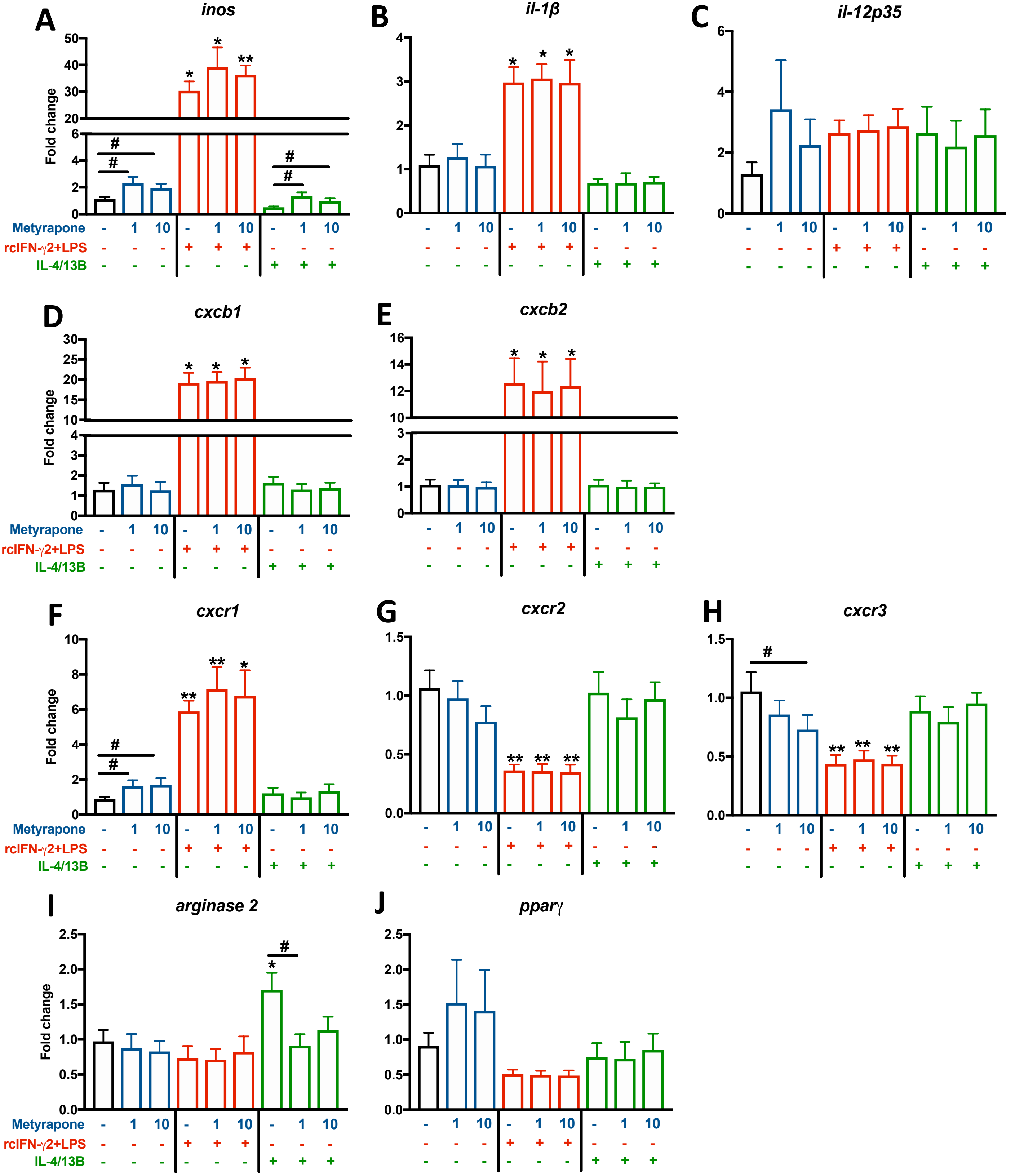

Supplementary Figure 2. Changes in the expression of pro-inflammatory molecules (**A-E**), CXC chemokine receptors (**F-H**), arginase 2 (**I**) and peroxisome proliferator-activated receptor gamma (PPARγ) (**J**) in the head kidney monocytes/macrophages. Cells were *in vitro* pretreated for 1 h with inhibitor of cortisol synthesis/conversion - metyrapone (1 μM, 10 μM) or vehicle (DMSO) and next for 48 h incubated with combination of lipopolysaccharide (30 μg/mL) and recombinant carp interferon gamma2 (100 ng/mL, rcIFN-γ2+LPS), danio recombinant IL-4 (100 ng/mL) or culture medium. Changes in gene expression are shown as x-fold increase compared to control cells cells and they were standardized for the housekeeping gene 40S ribosomal protein s11. Averages and S.E (n=5-6). Stars indicate significant differences between control and stimulated cells (*p ≤ 0.05, ** p ≤ 0.01). Number signs indicate significant differences between control cells and cells treated with metyrapone (#p ≤ 0.05).

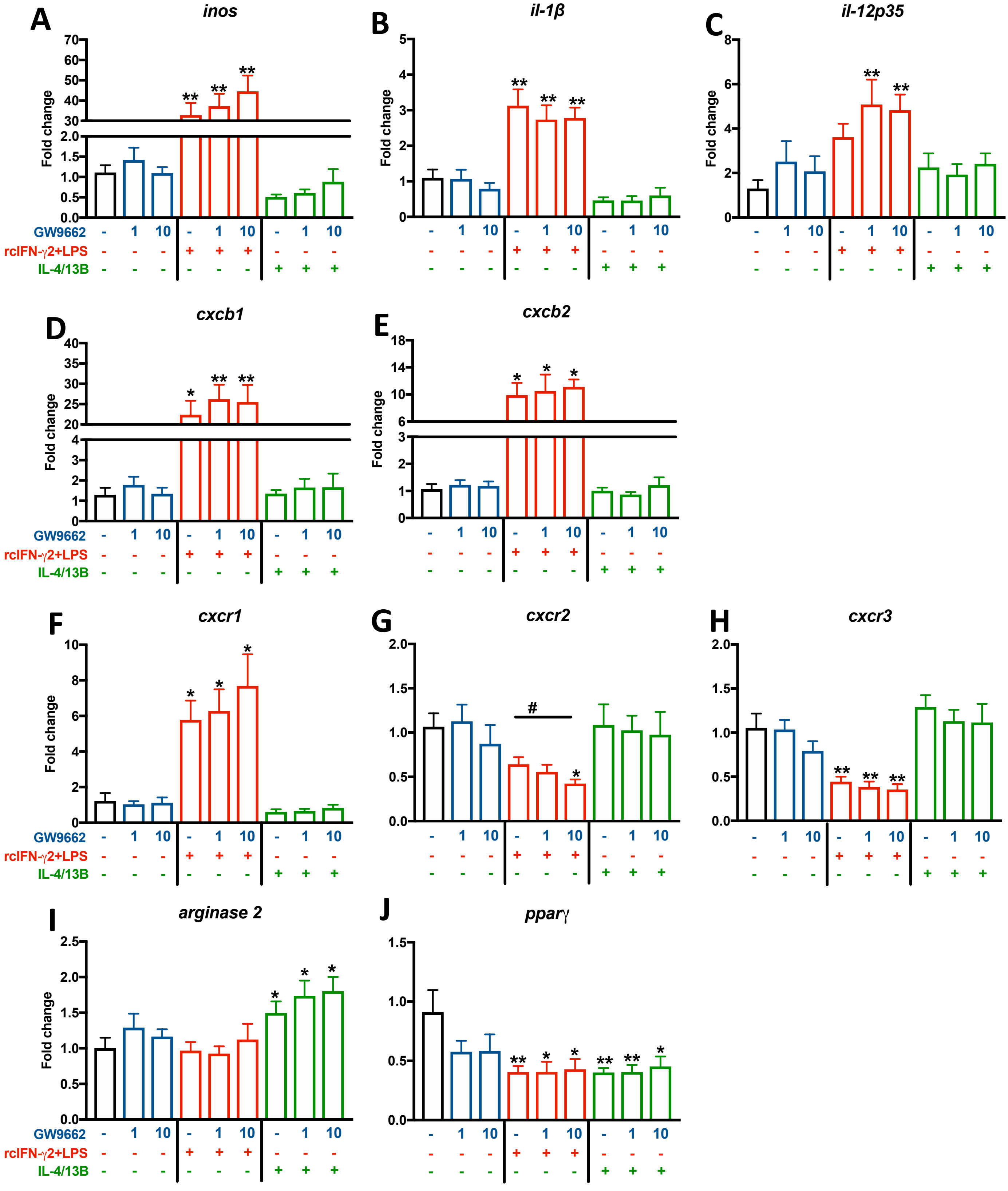

Supplementary Figure 3. Changes in the expression of pro-inflammatory molecules (**A-E**), CXC chemokine receptors (**F-H**), arginase 2 (**I**) and peroxisome proliferator-activated receptor gamma (PPARγ) (**J**) in the head kidney monocytes/macrophages. Cells were *in vitro* pretreated for 1 h with the selective PPARγ antagonist - GW9662 (1 μM, 10 μM) or vehicle (DMSO) and next, incubated for 48 h with a combination of lipopolysaccharide (30 μg/mL) and recombinant carp interferon gamma2 (100 ng/mL, rcIFN-γ2+LPS), danio recombinant IL-4 (100 ng/mL) or culture medium. Changes in gene expression are shown as x-fold increase compared to control cells and they were standardized for the housekeeping gene 40S ribosomal protein s11. Averages and S.E (n=5-6). Stars indicate significant differences between control and stimulated cells (*$p \leq 0.05$, ** $p \leq 0.01$). Number signs indicate significant differences between control cells and cells treated with GW9662 (#$p \leq 0.05$).

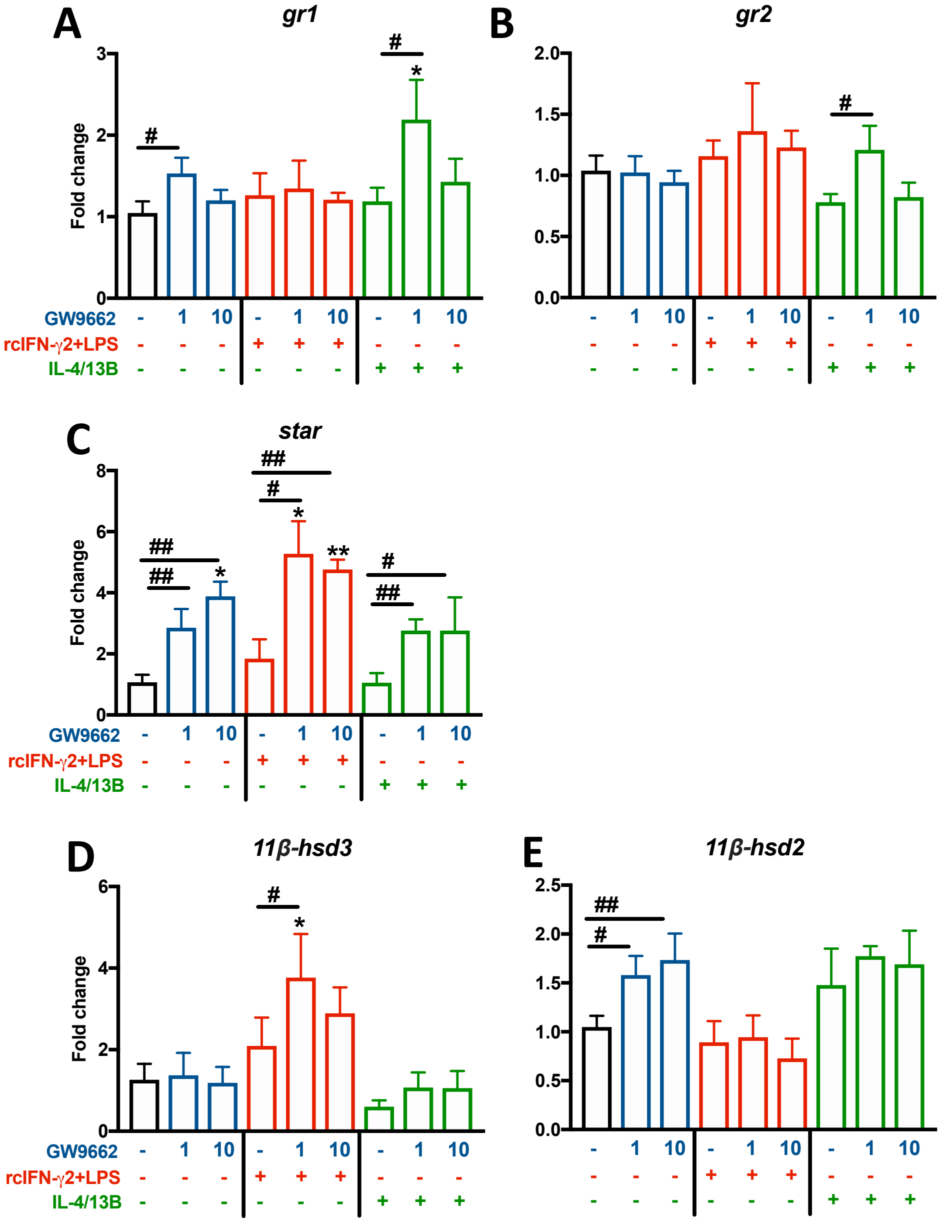

Supplementary Figure 4. Changes in the expression of glucocorticoid receptors (**A, B**) and molecules involved in the synthesis (**C**) and conversion of cortisol (**D, E**) in the head kidney monocytes/macrophages. Cells were *in vitro* pretreated for 1 h with selective PPARγ antagonist - GW9662 (1 μM, 10 μM) or vehicle (DMSO) and next for 48 h incubated with a combination of lipopolysaccharide (30 μg/mL) and recombinant carp interferon gamma2 (100 ng/mL, rcIFN-γ2+LPS), danio recombinant IL-4 (100 ng/mL) or culture medium. Changes in gene expression are shown as x-fold increase compared to control cells and they were standardized for the housekeeping gene 40S ribosomal protein s11. Averages and S.E (n=5-6). Stars indicate significant differences between control and stimulated cells (*$p \leq 0.05$, ** $p \leq 0.01$). Number signs indicate significant differences between control cells and cells treated with GW9662 (#$p \leq 0.05$, ## $p \leq 0.01$).

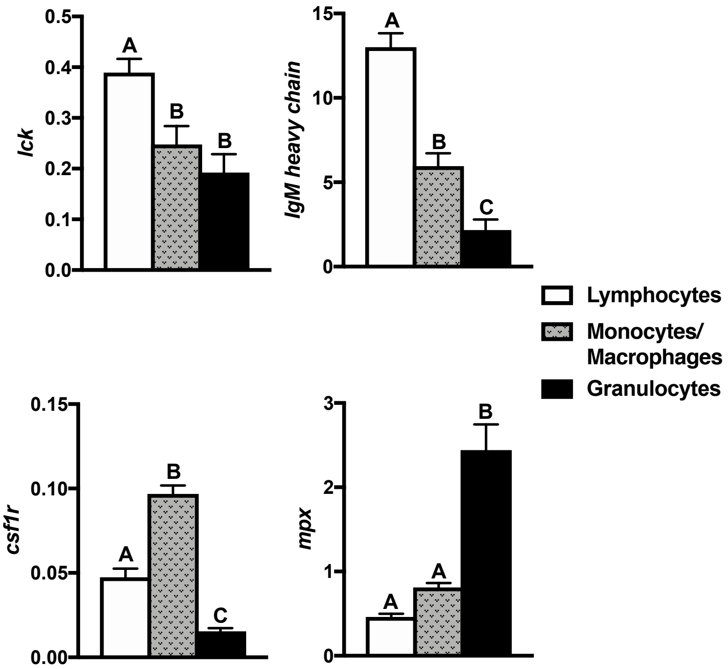

Supplementary Figure 5. Constitutive gene expression of *lck, IgM heavy chain, csf1r* and *mpx* in carp leukocytes from head kidney (lymphocytes - 1.02 g/cm^3, monocytes/macrophages - 1.06 g/cm^3 and neutrophilic granulocytes – 1.07 g/cm^3). Constitutive gene expression was determined by quantitative RT-PCR and expressed relative to expression of the 40S ribosomal protein s11 gene. Averages and S.E (n=5). Mean values not sharing letters are statistically different according ANOVA between different populations.

A

```
DaniorerioIL-4/13B  MRTFLLLVLTLVPVSESKLKIDEILLMEIIQSVNGILNGKGEKMDLDQFIPDIYETGHYS
CarpIL-4/13A        MKTILLLAFAVLVSSTPVENDDKILLGELIDELN--REVKRFSNDITEIFLAELEMNGCK
                    *:*:***.::::  * .  : *:*** *:*..:*   : *  . *: :::    * .  .

DaniorerioIL-4/13B  KKTLCQAGMALKGKIPSRS-----------HLQRQLNAYAAYTGTGNCSVSTSGECTMKE
CarpIL-4/13A        GEFFCQAEHELKEKVSGLSGAKFEHFRTDKKLMRNLNGYNKRHVKTCKPADKDQEILLHV
                     : :***   ** *:.. *           :* *:**.*     .   ..... *   ::

DaniorerioIL-4/13B  FLEKTKACCHYLYSAQRT
CarpIL-4/13A        FLRNLLTCAKRVYSQPK-
                    **.:  :*.: :**  :
```

B

```
DaniorerioIL-4/13B  MRTFLLLVLTLVPVSESKLKIDEILLMEIIQSVNGILNGKGEKMDLDQFIPDIYETGHYS
CarpIL-4/13B        MRTFMLLVLTIVAVTGIKHKAGQILLMEIIDDVKQILN-QSSSTNLNQFVIDVFPPVGCS
                    ****:*****:*.*:  * * .:*******:.*: *** :... :*:**: *:: .   *

DaniorerioIL-4/13B  KKTLCQAGMALKGKIPSRSHLQRQLNAYAAYTGTGNCSVSTSGECTMKEFLEKTKACCHY
CarpIL-4/13B        EKHICQAAMVMMNTELSHSMLHRRLFAYANYSGHLHCNVTASEEHRMDVFLEKIKDCCKA
                    :* :***.*.: ..  *:* *:*:* *** *:*  :*.*::* *  *. **** * **:

DaniorerioIL-4/13B  LYSAQRT-
CarpIL-4/13B        QYSKPLKQ
                     **   .
```

Supplementary Figure 6. Alignment of the amino acid sequence of zebrafish (*Danio rerio*) interleukin 4 (IL-4, CAL48253) and sequences of the interleukin 4/13A (IL-4/13A, BAM09149.1) **(A)** and interleukin 4/13B (IL-4/13B, BAL43179.1) **(B)** of common carp (*Cyprinus carpio* L.). Identical residues are marked with (*), conserved residues with (:) and similar residues with (.). Recombinant IL-4 sequence - shaded.

Supplementary Table 1. Constitutive gene expression of pro-inflammatory molecules, CXC chemokines and their receptors, anti-inflammatory mediators, PPARγ, GR and molecules involved in the synthesis/conversion of cortisol in the head kidney monocytes/macrophages. Cells were *in vitro* treated with combination of lipopolysaccharide (30 μg/mL) and recombinant carp interferon gamma2 (100 ng/mL, rcIFN-γ2+LPS), recombinant IL-4/13B (100 ng/mL) or culture medium (CTR). Basal gene expressions were standardized for the housekeeping gene 40S ribosomal protein s11. Averages and S.E (n=5-6). Mean values not sharing letters are statistically different.

**time**	**6 h**			**24 h**			**48 h**		
**gene**	**CTR**	**rcIFN-γ2+LPS**	**IL-4/13B**	**CTR**	**rcIFN-γ2+LPS**	**IL-4/13B**	**CTR**	**rcIFN-γ2+LPS**	**IL-4/13B**
inos	0.123±0.036<br>a	8.878±2.190<br>b	0.104±0.036<br>a	0.124±0.063<br>a	6.6487±1.837<br>bc	0.213±0.097<br>a	0.33±0.147<br>a	3.825±0.897<br>ac	0.072±0.019<br>a
il-1β	0.495±0.101<br>a	7.983±1.297<br>b	2.288±0.543<br>a	0.346±0.120<br>a	1.103±0.221<br>a	0.292±0.115<br>a	0.941±0.378<br>a	0.323±0.078<br>a	0.268±0.078<br>a
il-12p35	0.001±0.0003<br>ac	0.227±0.039<br>b	0.001±0.0001<br>ac	0.030±0.015<br>ac	0.042±0.008<br>ac	0.066±0.039<br>ac	0.257±0.098<br>b	0.049±0.013<br>c	0.139±0.067<br>abc
ifn-γ2	0.014±0.008<br>a	0.025±0.017<br>a	0.006±0.002<br>a	0.286±0.088<br>a	0.317±0.062<br>a	0.759±0.246<br>b	0.194±0.026<br>a	0.238±0.041<br>a	0.231±0.077<br>a
cxcb1	0.011±0.005<br>a	0.174±0.008<br>b	0.007±0.004<br>a	0.018±0.007<br>a	0.627±0.191<br>c	0.07±0.037<br>a	0.014±0.003<br>a	0.317±0.079<br>bc	0.016±0.002<br>a
cxcb2	0.059±0.016<br>a	3.348±1.311<br>b	0.055±0.005<br>a	0.283±0.061<br>a	2.191±0.478<br>bc	0.441±0.134<br>a	0.31±0.124<br>a	0.868±0.103<br>ac	0.156±0.016<br>a
cxcl8_l1	0.152±0.029<br>a	0.338±0.058<br>b	0.275±0.080<br>abc	0.324±0.081<br>ab	0.092±0.016<br>ac	0.200±0.034<br>abc	0.348±0.068<br>b	0.154±0.039<br>ac	0.154±0.009<br>abc
cxcl8_l2	0.064±0.024<br>a	0.056±0.014<br>a	0.071±0.025<br>a	0.287±0.087<br>b	0.192±0.056<br>b	0.261±0.098<br>b	0.036±0.009<br>ab	0.046±0.015<br>ab	0.048±0.016<br>ab
cxcr1	0.352±0.052<br>a	0.219±0.104<br>a	0.111±0.062<br>a	0.146±0.045<br>a	0.511±0.174<br>ab	0.091±0.033<br>a	0.037±0.013<br>a	1.167±0.452<br>b	0.36±0.008<br>a
cxcr2	0.021±0.004<br>ab	0.016±0.008<br>ab	0.010±0.003<br>a	0.029±0.013<br>ab	0.018±0.011<br>ab	0.060±0.017<br>b	0.034±0.017<br>ab	0.017±0.009<br>a	0.017±0.005<br>a
cxcr3	0.032±0.005<br>a	0.024±0.005<br>a	0.019±0.004<br>a	0.073±0.032<br>a	0.086±0.035<br>a	0.075±0.039<br>a	0.077±0.024<br>a	0.017±0.006<br>a	0.100±0.012<br>a
arginase 1	0.001±0.001<br>a	0.002±0.001<br>a	0.001±0.0002<br>a	0.001±0.0004<br>a	0.002±0.001<br>a	0.001±0.0003<br>a	0.003±0.001<br>a	0.001±0.0003<br>a	0.002±0.0004<br>a
arginase 2	0.299±0.055<br>a	0.286±0.044<br>a	1.292±0.320<br>b	0.243±0.106<br>a	0.350±0.062<br>a	1.289±0.241<br>b	0.590±0.156<br>a	0.465±0.101<br>a	0.888±0.149<br>ab
il-10	0.015±0.006<br>a	0.010±0.002<br>a	0.008±0.002<br>a	0.031±0.010<br>a	0.020±0.004<br>a	0.117±0.022<br>a	0.259±0.133<br>ab	0.165±0.130<br>a	0.579±0.154<br>b
mmp9	1.275±0.143<br>a	1.822±0.283<br>a	2.845±0.763<br>a	10.720±3.814<br>a	4.250±0.751<br>a	7.776±1.342<br>a	28.474±8.771<br>b	7.699±1.069<br>a	23.335<br>b
pparγ	0.042±0.011<br>a	0.006±0.002<br>a	0.011±0.002<br>a	0.040±0.015<br>a	0.018±0.006<br>a	0.016±0.007<br>a	0.047±0.029<br>a	0.030±0.013<br>a	0.005±0.001<br>a
gr1	0.067±0.01<br>a	0.106±0.023<br>ab	0.072±0.016<br>a	0.052±0.013<br>a	0.224±0.062<br>b	0.110±0.037<br>ab	0.237±0.02<br>b3	0.193±0.029<br>ab	0.196±0.021<br>ab
gr2	0.252±0.094<br>a	0.251±0.108<br>a	0.103±0.026<br>a	0.159±0.040<br>a	0.331±0.110<br>a	0.136±0.048<br>a	0.332±0.036<br>a	0.422±0.049<br>a	0.206±0.024<br>a
mr	0.011±0.005<br>a	0.003±0.001<br>a	0.004±0.002<br>a	0.003±0.001<br>a	0.004±0.001<br>a	0.004±0.001<br>a	0.008±0.002<br>a	0.005±0.001<br>a	0.005±0.001<br>a
star	0.008±0.002<br>ab	0.011±0.003<br>a	0.004±0.001<br>ab	0.004±0.001<br>a	0.003±0.001<br>a	0.002±0.001<br>a	0.003±0.001<br>a	0.003±0.001<br>a	0.004±0.002<br>a
11β-hsd3	0.003±0.001<br>ab	0.004±0.001<br>ab	0.003±0.001<br>ab	0.002±0.001<br>a	0.005±0.002<br>b	0.002±0.001<br>a	0.004±0.001<br>ab	0.006±0.001<br>ab	0.003±0.001<br>ab
11β-hsd2	0.006±0.003<br>a	0.005±0.003<br>a	0.042±0.015<br>b	0.094±0.041<br>abc	0.054±0.018<br>abc	0.256±0.135<br>c	0.119±0.024<br>abc	0.083±0,046<br>abc	0.273±0.134<br>c

Supplementary Table 2. Primers used for quantitative real-time PCR analysis.

Gene	Primer forward (5`-3`)	Primer reverse (5`-3`)	Acc. No	μM
40s11	CCGTGGGTGACATCGTTACA	TCAGGACATTGAACCTCACTGTCT	AB012087	1
inos	AACAGGTCTGAAAGGGAATCCA	CATTATCTCTCATGTCCAGAGTCTCTTCT	AJ242906	1
il-1β	AAGGAGGCCAGTGGCTCTGT	CCTGAAGAAGAGGAGGCTGTCA	AJ245635	1
il-12p35	TGCTTCTCTGTCTCTGTGATGGA	CACAGCTGCAGTCGTTCTTGA	AJ580354	1
cxcb1	GGGCAGGTGTTTTTGTGTTGA	AAGAGCGACTTGCGGGTATG	AB082985	1.125
cxcb2	AGGCAGGTGCTTCTGTGCTGACA	TTCATGCATTTCCGCTCTGCGCT	JN104598	1.125
ifn-γ2	TCTTGAGGAACCTGAGCAGAA	TGTGCAAGTCTTTCCTTTGTAG	AM168523	1
cxcl8_l1	CTGGGATTCCTGACCATTGGT	GTTGGCTCTCTGTTTCAATGCA	AJ421443	1.125
cxcl8_l2	TCACTTCACTGGTGTTGCTC	GGAATTGCTGGCTCTGAATG	AB470924	1.125
cxcr1	GCAAATTGGTTAGCCTGGTGA	AGGCGACTCCACTGCACAA	AB010468	1.125
cxcr2	TATGTGCAAACTGATTTCAGGCTTAC	GCACACACTATACCAACCAGATGG	AB010713	1.125
cxcr3	CACTCTGTCACGCCACTCGTAGG	TGTCAATGACCCCAAGCATCTGC	HE584636	1.125
arginase 1	TGAGGAGCTTCAGCGGATTAC	CCTATTATTCCCACGCAGTGATG	AJ871264	1
arginase 2	GGAGACCTGGCCTTCAAGCATCT	CTGATTGGCACGTCCAACT	AJ618955	1
il-10	CGCCAGCATAAAGAACTCGT	TGCCAAATACTGCTCGATGT	AB110780	1
mmp-9	ATGGGAAAGATGGACTGCTG	TCAAACAGGAAGGGGAAGTG	AB057407	2.25
pparγ	ACCCTCATCTCCTACGGTCA	GGGCTCCATCATTTCACAGA	FJ849064.1	1
gr1	GACTTACCTGACTCCCTATCTGAC	GCTTCCACCATCTGCTGC	AJ879149	1.5
gr2	GGAGAACAACGGTGGGACTAAAT	GGCTGGTCCCGATTAGGAA	AM183668	1.5
mr	TTCCCTGCAGAACTCAAAGGA	ACGGACGGTGACAGAAACG	AJ783704	2
star	GTGGAACCCCAATGTCAAAC	ACAGGTGGGTCCATTCTCAG	FJ490418	1
cyp11b1	CCCTGGAAGGTCAGTGTTGT	GGTGGGGTTTGGAGATAAGG	XM_019123804.1	2
11β-hsd3	GCACTCAATGGTTTCTTTGGA	GCTCCAGCTTCGATAATGTG	XM_019107831	2
11β-hsd2	TACGCAAAAACAGCCAATGA	GTAGTATCGCACCTGGGGTTG	XM_019069125	2
mpx	GTGGTCGTGTCGGTCCTCTT	GATGCCTTTTGTTTGGTGGTG	AB429306	1
lck	CAAGTGGACGGCACCTGA	GCAATCACCTCTGGGTTTGT	XM_019077902.1	1
IgM heavy chain	CACAAGGCGGGAAATGAAGA	GGAGGCACTATATCAACAGCA	AB004105	1
csfr1	CAGGAAACCGGCCACTACA	CCCATCTCACCATCGCTACA	AB526448	1
